# Enhancing Manufacturability of SU-8 Piezoelectric Composite Films for Microsystem Applications

**DOI:** 10.3390/mi15030397

**Published:** 2024-03-14

**Authors:** Irma Rocio Vazquez, Zeynel Guler, Nathan Jackson

**Affiliations:** 1 Department of Mechanical Engineering, University of New Mexico, Albuquerque, NM 87131, USA; 2 Nanoscience and Microsystems Engineering, University of New Mexico, Albuquerque, NM 87131, USA

**Keywords:** piezoelectric, thin films, microelectromechanical systems, SU-8, composite

## Abstract

Piezoelectric thin films are extensively used as sensing or actuating layers in various micro-electromechanical systems (MEMS) applications. However, most piezoelectrics are stiff ceramics, and current polymer piezoelectrics are not compatible with microfabrication due to their low Curie Temperature. Recent polymer-composite piezoelectrics have gained interest but can be difficult to pattern. Photodefinable piezoelectric films could resolve these challenges by reducing the manufacturability steps by eliminating the etching process. But they typically have poor resolution and thickness properties. This study explores methods of enhancing the manufacturability of piezoelectric composite films by optimizing the process parameters and synthesis of SU-8 piezo-composite materials. Piezoelectric ceramic powders (barium titanate (BTO) and lead zirconate titanate (PZT)) were integrated into SU-8, a negative epoxy-based photoresist, to produce high-resolution composites in a non-cleanroom environment. I-line (365 nm) light was used to enhance resolution compared to broadband lithography. Two variations of SU-8 were prepared by thinning down SU-8 3050 and SU-8 3005. Different weight percentages of the piezoelectric powders were investigated: 5, 10, 15 and 20 wt.% along with varied photolithography processing parameters. The composites’ transmittance properties were characterized using UV-Vis spectroscopy and the films’ crystallinity was determined using X-ray diffraction (XRD). The 0–3 SU-8/piezo composites demonstrated resolutions < 2 μm while maintaining bulk piezoelectric coefficients d_33_ > 5 pm V^−1^. The films were developed with thicknesses >10 μm. Stacked layers were achieved and demonstrated significantly higher d_33_ properties.

## 1. Introduction

Integrating ceramic nano- and micro-powders into polymeric matrices opens the door to exploring new materials with unique properties whose performance can be regulated [1,2]. The development of new functional and multifunctional thin films is of particular interest with regard to microelectromechanical systems (MEMS) devices, as they rely on these materials for actuation or sensing applications. Materials that have piezoelectric properties are widely used in MEMS as they have the capability to convert mechanical energy into electrical energy, and vice versa. Polymer MEMS are increasingly in demand because of their elasticity properties, relatively low cost to manufacture, and increased biocompatibility [2,3]. However, most polymers are not “smart” materials, meaning they have no functionality. Polyvinylidene fluoride (PVDF) is a known piezoelectric polymer that has been used extensively, but its low Curie Temperature (approximately 100 °C) makes integration into standard MEMS fabrication challenging [4,5,6].

Piezoelectric polymers are increasingly in demand because of the numerous possible applications [3,7,8]. Various methods have been investigated to create new piezoelectric polymers that are MEMS-compatible; including modifying semi-crystalline polymers to have piezoelectric properties such as parylene [9,10,11,12], creating stacked layers of alternating piezoelectric and polymer films [13,14,15,16,17], and creating composite materials consisting of a polymer matrix with piezoelectric particles. Combining piezoelectric particles into a polymeric matrix can result in multi-functional composites that combine piezoelectric and ferroelectric properties, while still maintaining the mechanical properties of the polymer matrix [18,19,20]. Mechanical properties of polymers offer benefits in a wide range of applications including, but not limited to, low-frequency vibrational energy harvesters [21,22,23,24,25] and biomedical sensors [26,27,28,29].

Thin-film polymer piezoelectric composites have been widely investigated over the past decade [1]. Composites with an 0–3 structure, where particles are dispersed randomly throughout the matrix, are common due to manufacturing benefits, but often have problems with particle orientation [1,30]. Integration of 0–3 composites is challenging for MEMS applications that require patterning at the micro-scale; using non-photodefinable materials often leads to complex etching methods being needed for each composite material [31]. Using photoresist or photodefinable polymers as the matrix for the composites provides patternability, which is desired for MEMS integration. These composites can then have multifunctional characteristics, depending on the particles used, that can convert MEMS-compatible polymers into “smart” materials.

The relationship between the amount of powder material in terms of weight percentage (wt.%) and photopatternability in photoresist matrices has been previously investigated [22,32,33,34] and it was determined that resolution was significantly reduced as the wt.% of particles increased, as this increase affected the transmittance of light through the polymer medium. However, functionality typically increases with particle concentration. Nonetheless, this is not a straightforward relationship, as various factors affect the final piezoelectric performance of the composite. The photopatternability of these composite polymers could increase manufacturability, making integration with silicon-based microfabrication more accessible [35].

SU-8 is a negative epoxy–based photoresist that is commonly used in high-aspect-ratio micro-structures and numerous MEMS devices [36]. SU-8’s high compatibility with MEMS fabrication, due to its high thermal properties and photopatternability, for instance, makes it in an ideal polymer matrix material. Previously, 0–3 SU-8 composites with Zinc Oxide (ZnO), Barium Titanate (BTO), Lead Zirconate Titanate (PZT), Lead Magnesium Niobate/Lead Titanate (PMN-PT) have been investigated for piezoelectric performance [22,32,33,37,38,39,40,41]. Carbon and Ag powders have been integrated to increase electrically conductive properties [42,43]. These SU-8 composites have also been applied to microdevices, such as piezoelectric-based cantilevers [37,40,44,45], flexible nanogenerators [32], and acoustic resonators/transducers [41]. However, previous results have been limited by the minimum feature size that could be patterned (>50 μm) and the film thickness used (<5 μm). Typical academic research exposure tools use broadband wavelengths that consist of i-line, h-line, and g-line exposures which affect the crosslinking of the negative resist. In addition, previous 0–3 composites often require long manufacturing times due to processes that include probe sonication for homogenous mixing. A systematic investigation into the optimization of the photolithography process has not been previously reported. Other MEMS-compatible photodefinable polymers have also being investigated, including polyimide [46], but SU-8 is more widely investigated.

In this paper, the authors investigate methods of increasing the MEMS manufacturability of SU-8 0–3 piezoelectric composites. The goal of the study was to optimize photolithography process parameters and mixing methods to enhance critical dimensions of structures to the micro-scale while increasing thickness of the layers. The study involved investigating two different types of SU-8 along with three different piezoelectric nanoparticles (BTO, PZT, and ZnO) with varying concentrations: 5, 10, 15 and 20 wt.%. The composite films were created by using a speed mixer instead of a probe sonicator to reduce manufacturing time and increase homogeneity. A specified i-line (365 nm) exposure tool was used instead of a broadband exposure tool, and the films were all made in a normal lab environment under yellow lights, so no cleanroom was needed. The paper illustrates the ability to deposit and pattern films onto various substrates (Si, Al, glass, and polymers). The study also investigated methods of increasing the piezoelectric properties and thickness by creating stacked layer structures, and the piezoelectric performance was demonstrated by tapping the film to light up an LED.

## 2. Materials and Methods

### 2.1. Fabrication of Composite Films

Various types of SU-8 thin-film composites were prepared in a traditional lab environment. SU-8 3050 and SU-8 3005 (Kayaku Advanced Materials, Westborough, MA, USA) were used as the base material, but were reduced to produce SU-8 3005 and SU-8 3000.5 using cyclopentanone thinner using Equation (1) [32]; throughout the paper, 3005 and 3000.5 refer to the thinned version. Where $V_{T}$ represents the volume of SU-8 thinner, *V_SU_*_8_ is the volume of SU-8, $\rho_{SU8}$ is the density of the starting SU-8, $\rho_{T}$ is the density of the thinner, $S_{RI}$ is the % solid content of the starting SU-8, and $S_{RN}$ is the % solid content of the final SU-8. Thinner was used to reduce the base material to create SU-8 3005 and 3000.5 instead of just using commercial 3005 and 3000.5 because mixing the particles with the thinner helped to prevent agglomeration of the particles.

(1) $$V_{T}=V_{SU8}\frac{\rho_{SU8}}{\rho_{T}}\left(\frac{S_{RI}}{S_{RN}}-1\right)$$

**Figure 1:**
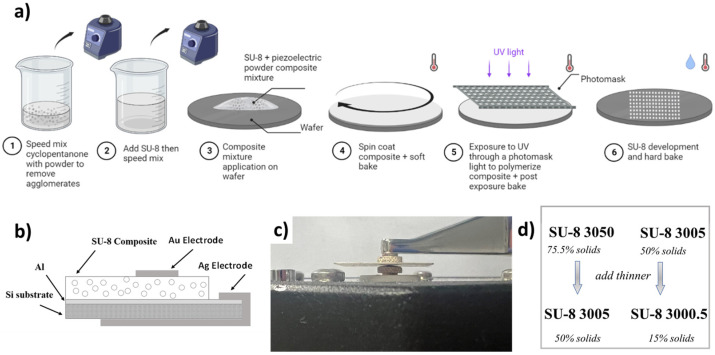
(**a**) Schematic illustrating the process steps to produce the SU-8 composites (**b**) Cross-section schematic of the structured layers used for piezoelectric poling and characterization (**c**) Image of the piezometer characterization setup (**d**) Types of SU-8 used as the base material.

Initially, three types of ceramic piezoelectric particles were used to make the composite materials: Barium Titanate (BTO) (Nanostructured & Amorphous Materials, Inc., Katy, TX, USA, average particle size: 200 nm), lead zirconate titanate (Pb(Zr_0.52_T_0.48_)O_3_) (PZT) (American Elements, Los Angeles, CA, USA, average particle size: ≤5 μm), and Zinc Oxide (ZnO) (SkySpring Nanomaterials Inc., Houston, TX, USA, average particle size: 60 nm). The PZT particles were milled down to an average particle size of 300 nm (FlackTek Speed Mixer and milling). The BTO and PZT particles were calcined at a temperature of 750 °C in a furnace oven under vacuum for 3 h [47]. The overall process for creating the composite films is illustrated in Figure 1. The particles were first mixed with the SU-8 thinner to prevent agglomeration. The nanoparticles were measured and mixed to have a final SU-8 resin concentration of 5, 10, 15 and 20 wt.%. The particles and thinner were mixed using a speed mixer (FlackTek Speed Mixer, Landrum, SC, USA) at 1000, 2500, and 3000 rpm for 1 min each with 5 min resting time between cycles to prevent heating of the thinner. Mixing conditions used by other researchers required long mixing times due to using probe-sonication and ice to prevent heating. However, using the speed mixer reduced preparation time, making manufacturability less time-consuming. The speed mixing process resulted in a homogenous mixture of thinner and piezoelectric particles.

After the thinner was mixed with the particles, the mixture was added to the initial SU-8 base material (3050 and 3005). Adding the thinner resulted in the creation of SU-8 3005 and 3000.5 films. The SU-8/thinner/particle mixture was then mixed using the speed mixer with the same mixture protocol as used with the thinner/particles. Eliminating heating is critical to prevent evaporation of the solvent. The SU-8/composite mixture was dispensed on various substrates to illustrate the potential integration onto bare Si, glass, Al coated Si, and polydimethylsiloxane (PDMS). The default substrate used for characterization was Al coated on Si, which consisted of (100) Si wafer with sputtered (300 nm) Al layer. All substrates were cleaned prior to dispensing the mixture using a Tergeo Plasma Cleaner (PIE Scientific, Union City, CA, USA) in O_2_ plasma to remove any organic contaminants; the substrates were also cleaned in solvent baths of acetone, methanol, and isopropyl alcohol, and dried with $N_{2}$ gas, followed by a dehydration bake at 125 °C for 5 min. To improve adhesion, OmniCoat (Kayaku, Westborough, MA, USA) was spin coated on the substrates at 3000 rpm (500 rpm for 5 s with an acceleration of 100 rpm/s, followed by 3000 rpm casting speed for 30 s with an acceleration of 300 rpm/s), and then baked at 200 °C on a hot plate for 1 min. Then, the substrates were allowed to cool down to room temperature prior to dispensing the mixture on the substrate.

As shown in Figure 1a, the composite mixtures were spin coated using an initial speed of 500 rpm for 5 s with an acceleration of 100 rpm/s, followed by different casting (1000, 2000, 3000, 4500, and 6000 rpm) speeds to alter the thickness of the films for 30 s with an acceleration of 300 rpm/s. The different spin speeds produced different thickness due to the varying properties of the mixtures with different concentrations. Characterization was performed on films with a casting speed of 3000 rpm unless specifically specified. The films underwent a standard photolithography process to pattern the composite films. The next step consisted of a soft bake to evaporate the solvents of the photoresist at 95 °C at different soft-bake times, depending on the powder used and the concentration of the film. The optimal processing parameters for the composites were found by testing different parameters for each respective concentration. The UV-exposure was performed on an i-line (365 nm) LED exposure system (UV-KUB 2, Kloe, Saint-Mathieu-de-Tréviers, France) that had varying intensity capabilities. The composites were exposed with a 10 μm gap using a chrome/glass metrology photomask. The exposure was followed by a post-exposure bake (PEB) at 95 °C with varying time. The substrates were then submerged in SU-8 Developer to remove the soluble (unexposed) areas of the mixture. The samples were then hard baked at 150 °C for 10 min.

The photolithography steps were optimized for the various piezolectric composites, substrate, concentration, and thickness of the film. The optimization process consisted of varying each parameter and determining the minimum resolution dimensions. Stacks consisting of multiple layers of composite films were developed by repeating the fabrication process followed by poling of the entire stack. Multiple layer stacks of the composite films were developed to increase the overall thickness and to increase the overall piezolectric properties as the effective d_33_ = nd_33,f_ where n is the number of layers and d_33,f_ is the value of a single layer.

To prepare samples for piezoelectric characterization, the mixture was deposited on Al on Si substrates. Electrically conducting Ag ink (Ted Pella, Redding, CA, USA) was coated on the Al bottom layer and wrapped around to the bottom of the Si to create an interconnection to the backside of the substrate as shown in Figure 1b. The top electrode consisted of sputtered Au (100 nm) using a circular shadow mask. The samples were poled at 95 °C using a corona poling system (PK-C30kV-200C, PolyK, Philipsburg, PA, USA) at −18 kV for 10 min.

### 2.2. Characterization of Films

The piezoelectric (d_33,f_) properties were measured using a quasi-static Berlincourt method (Piezometer System PM 300, Piezotest, Singapore) with a resolution of <0.01 pC/N. The piezometer measures the bulk piezoelectric properties of the film, which is the property of interest to MEMS researchers. The measured properties are represented by d_33,f_ as the films remained on the Si substrate. The actual d_33_ values of the films will be higher and can be calculated based on the mechanical properties of films [48,49,50]; however, the reported d_33_ values in this paper refer to the measured d_33,f_ values.

To optimize resolution, the transmittance of the films was analyzed using a Cary 7000 Universal Measurement Spectrophotometer (Agilent, Santa Clara, CA, USA). Films containing three different particles (BTO, PZT, and ZnO) with various concentrations 5–20 wt.% were analyzed from 300–800 nm. The transmission measurements provided insight into the amount of i-line light (365 nm) that was able to penetrate the composite films, which ultimately determines the resolution and manufacturability of the films.

Crystallinity of the particles within the composite was measured using X-ray diffraction (XRD) (Malvern Panalytical, Malvern, UK). The films were analyzed at 45 kV and 40 mA using a 2-theta scan from 20–80°. XRD scans were performed before and after poling. Thickness of the films was measured using a profilometer (Veeco Dektak 8, Plainview, NY, USA), where the average of 10 scans was used to determine thickness as a function of spin speed and concentration. Resolution images were taken using a scanning electron microscope (SEM) (Phenom Pro X, Thermoscientific, Waltham, MA, USA).

## 3. Results and Discussion

### 3.1. Manufacturing of SU-8 Composites

**Figure 2:**
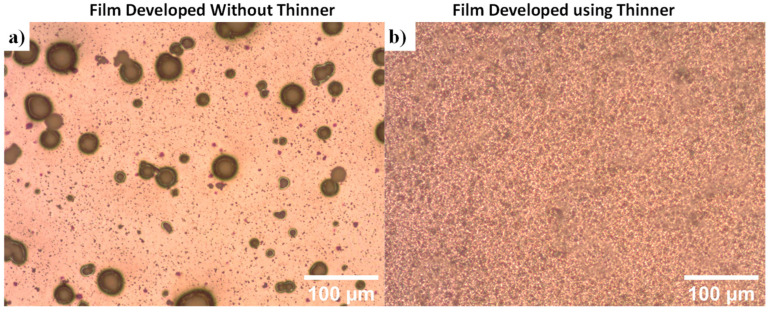
Optical images of SU-8/PZT composites (**a**) without the use of thinner and (**b**) with thinner.

Agglomeration of piezoelectric particles in a composite film can lead to reduced performance [31,51]. Images of films developed with and without thinner are illustrated in Figure 2. When the PZT particles were mixed directly into the SU-8 and then processed according to Figure 1 the films illustrated significant agglomeration of the particles resulting in large cluster of particles, where the piezoelectric properties were near zero due to poor orientation alignment. However, when the particles were mixed with thinner before being mixed with SU-8, the agglomeration significantly decreased (Figure 2b). Therefore, all films were made using the standard process illustrated in Figure 1, which included mixing particles with the thinner prior to mixing with SU-8.

**Figure 3:**
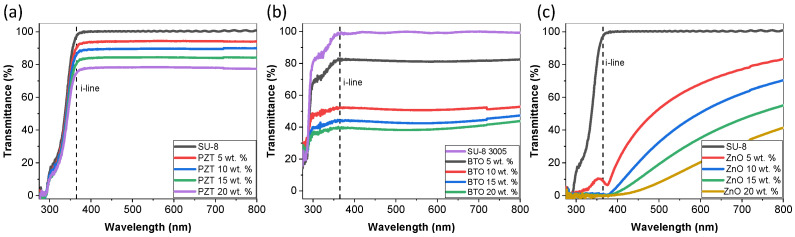
Transmission as a function of wavelength with varying particle concentration of 3005 SU-8 material spin coated at 3000 rpm (**a**) SU-8/PZT composite, (**b**) SU-8/BTO composite, and (**c**) SU-8/ZnO composite.

The transmission analysis as a function of wavelength for PZT, BTO, and ZnO composite films with varying concentration is shown in Figure 3. The transmittance is an important property as it determines patternability; adding particles to the SU-8 will decrease the transmittance, therefore blocking the UV exposure and preventing crosslinking of the SU-8, making the material soluble to developer. The films investigated were spin coated at 3000 rpm; other spin-speed films were investigated but there was no significant difference. The results illustrate a high transmission at 365 nm for SU-8/PZT films. Pristine SU-8 3005 had a transmittance of 98%, and as we increased the concentration of PZT the value decreased, which was expected, but even at 20 wt.% the transmittance was nearly 75%. Barium Titanate particles had a more significant impact as 20 wt.% film had a transmittance of 40%. However, ZnO composite films all had transmittance values of <10% at 365 nm. For this reason, ZnO composite films were not further investigated in this paper as patterning the samples using i-line exposure would require significant intensity. I-line exposure was used to enhance resolution capabilities which are dependent on wavelength. However, using h-line (405 nm) or g-line (436 nm) would be better for ZnO composites [32]. The transmittance results are likely due to variation in particle size and density of the particles as the concentrations were based on wt.%. Since the transmittance was not significantly different for various thicknesses (spin speeds) the exposure values are not likely to change significantly, although thicker films will require a slightly higher exposure dose.

**Figure 4:**
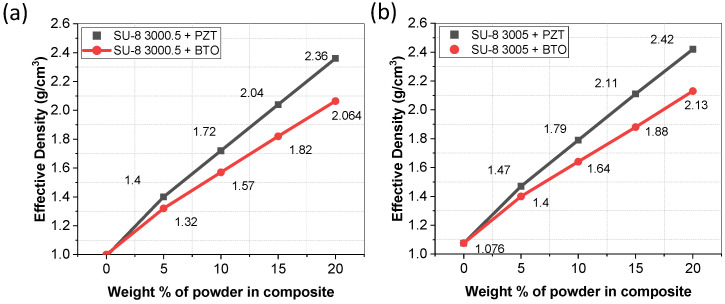
Density of composite films for (**a**) SU-8 3005 and (**b**) SU-8 3000.5 with various concentrations of PZT and BTO.

The density of the mixture will affect the thickness, ability to pattern the films, and is useful information for finite element modelling as it affects the mass. The density of the films was measured by weighing the mixture and dividing by the volume. The results shown in Figure 4, as expected, increase linearly as the particle concentration increases. The PZT/SU-8 mixture had a higher density due to PZT having a higher density of 8 g cm^−3^ compared to BTO (6 g cm^−3^). The densities of the pristine SU-8s agree with values from the company.

**Figure 5:**
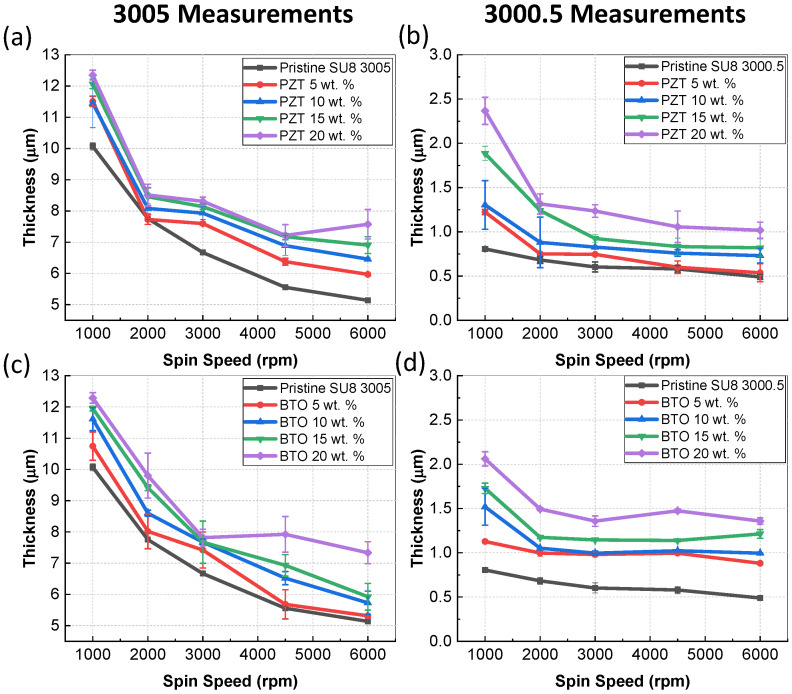
Average thickness as a function of final spin speed for various concentrations of composites (**a**) 3005 SU-8 with PZT particles, (**b**) 3000.5 SU-8 with PZT, (**c**) 3005 SU-8 with BTO, and (**d**) 3000.5 SU-8 with BTO the error bars represent the standard deviation.

The thickness of the films was measured using a profilometer to determine the effects of varying particle concentration. The results of the thickness of the film as a function of spin speeds is shown in Figure 5. In all cases, the pristine SU-8 agrees with values reported from the company, which means that the concentration of thinner using equation 1 gives an accurate representation of 3005 and 3000.5. All films had a decreased thickness as spin speed increased which was also expected due to spin theory. As we increased the concentration of the particles, the thickness increased, which was also expected as the density and viscosity increased. Thickness values were measured at various locations on the substrate, and there was a significant Increase in thickness on the edge due to edge bead, which was not included in the measurement and could be resolved by applying thinner on the edge. Composite materials made from PZT had a slightly higher thickness compared to films made from BTO with the same concentration due to the increased density of PZT films. Using SU-8 3000.5 sub-micron thicknesses could be achieved, while using 30005 resulted in thickness values > 12 μm for a single layer.

**Figure 6:**
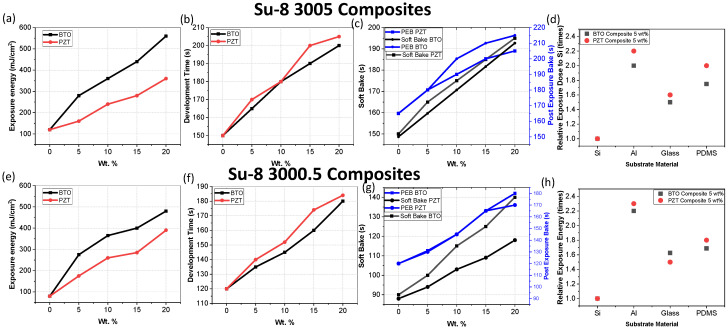
Optimal parameters for both SU-8 3000.5 and 3005 with varying concentrations of BTO and PZT on a Si substrate, (**a**,**e**) illustrate exposure energy, (**b**,**f**) illustrate development time, (**c**,**g**) illustrates soft bake and PEB time, and (**d**,**h**) illustrates the relative increase in exposure for patterning on different substrates.

Optimization of the processing parameters is critical for manufacturing the films. Figure 6 illustrates optimal exposure energy (a,e), development time (b,f), soft-bake time (c,g) and PEB (c,d). The optimal values were determined by visually inspecting the films to produce the best resolution structures. As expected from the transmittance results (Figure 3), BTO required higher exposure energy as it had lower transmittance, and the exposure required to pattern the films increased with increasing wt.% concentration. Development time, soft-bake time, and PEB time also increased with increasing wt.%. The SU-8 3005 required higher exposure, development time, soft-bake time, and PEB due to the increased thickness of the films. The values were optimized for an i-line exposure machine and thus will vary with g-line, h-line, or broadband exposure tools, but the trends should be similar. Figure 6d,h illustrate that the exposure energy should be adjusted depending on the substrate due based on their reflective properties.

### 3.2. Characterization of Composite Films

**Figure 7:**
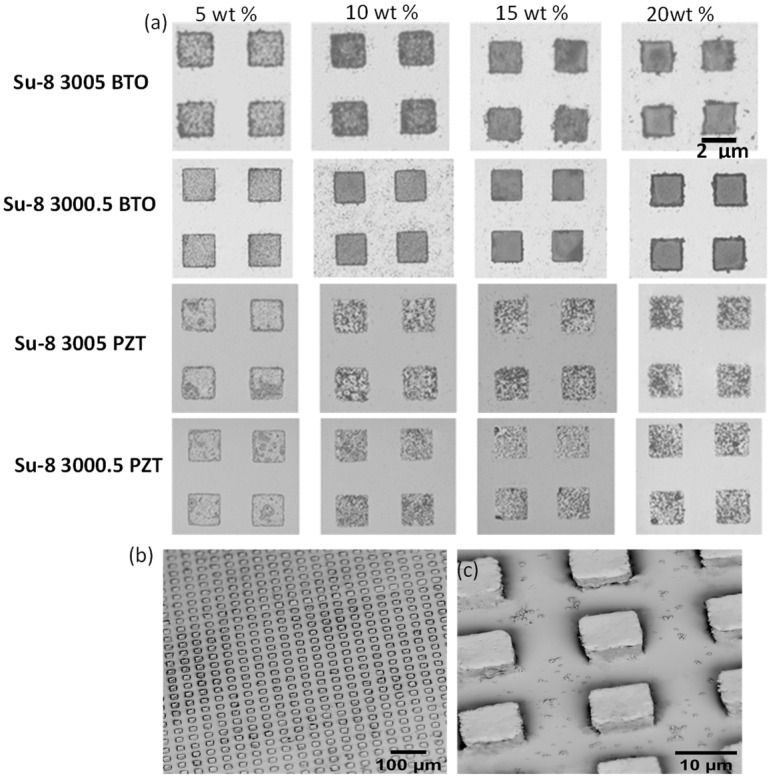
SEM images of patterned films of 30005 and 3000.5 with PZT and BTO at varying concentrations (**a**) top view, (**b**) array of patterned structures (BTO 20 wt.%) and (**c**) tilted structures showing the taper angle and thickness (BTO 20 wt.%).

Increasing resolution is often desired for MEMS applications and previous studies have demonstrated a resolution of 50–100 μm [32,33]. The resolution results for this study are shown in Figure 7. Using i-line exposure and optimizing the process parameters a resolution of <2 μm was achieved. The resolution could be achieved for various concentrations and thicknesses. However, PZT films had a smoother edge when compared to BTO samples, likely due to the transmittance. In all samples, the pattern is clearly visible with little to no SU-8 remaining in non-exposed areas. The increase in resolution compared to previous studies is believed to be due to the i-line exposure and optimized parameters, as previous studies from researchers used a traditional broadband exposure. Using a smaller and specific wavelength allowed the resolution to be reduced. The decreased resolution allows researchers to create small piezoMEMS structures. Previous research also had limitations due to using thicknesses of only a few microns, but we demonstrated the ability to pattern thick structures over 12 μm, which is significant for piezoelectric applications requiring higher actuation.

**Figure 8:**
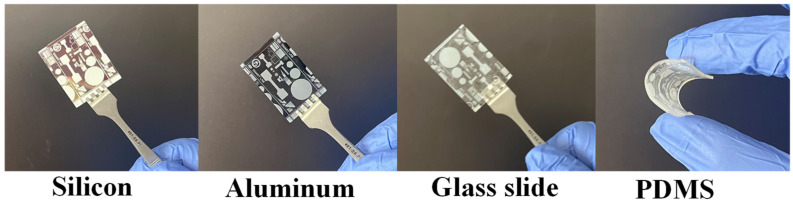
SU-8/PZT composite test structures deposited and patterned on various substrates.

The ability to deposit piezoelectric materials on various substrates is often desired for MEMS researchers. SU-8 (3005) with PZT particles at 20 wt.% were deposited and patterned according to the process parameters in Figure 6 on various substrates. The results are illustrated in Figure 8. The test structures were successfully deposited on Si, Al, glass, and PDMS (Sylgard 184). The resolutions achieved on the various substrates were similar to the results shown in Figure 7. The image illustrates the potential to bend or stretch the materials without any visible crack formation. However, SU-PZT structures will likely crack before the PDMS due to their more brittle mechanical properties, but the structures survived an acute bending test; future tests could involve a detailed reliability testing.

**Figure 9:**
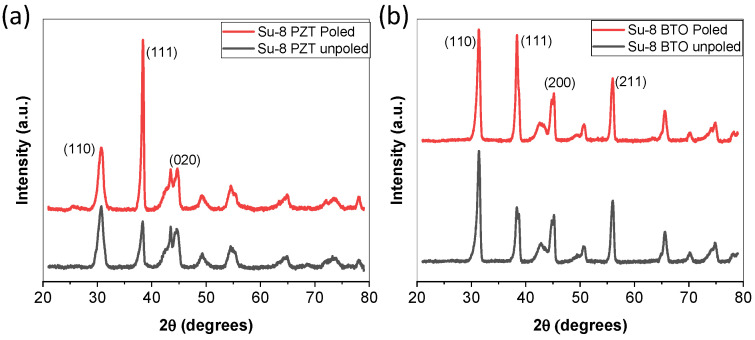
XRD results of SU-8 3005 composite films before and after poling, (**a**) PZT 20 wt.% and (**b**) BTO 20 wt.%.

The XRD results illustrated in Figure 9 demonstrate the integration of the ceramic PZT and BTO before and after poling. Before poling, the films illustrate a relatively high (111) and (110) textured crystallinity due to the particles. After poling, both films illustrated an increase in intensity and a decrease in full width half maximum (FWHM) in the (111) orientation, which is the orientation with high piezoelectric properties. The SU-8/PZT film had a (111) FWHM of 0.805° prior to poling and a value of 0.368° after poling. All other peaks had no significant changes to the FWHM. The SU-8/BTO had a reduction in FWHM in the (111) orientation from 0.798° to 0.6° after poling. Typically, the piezoelectric response are a function of crystallinity, so a lower FWHM value is likely to result in a better piezoelectric response.

**Figure 10:**
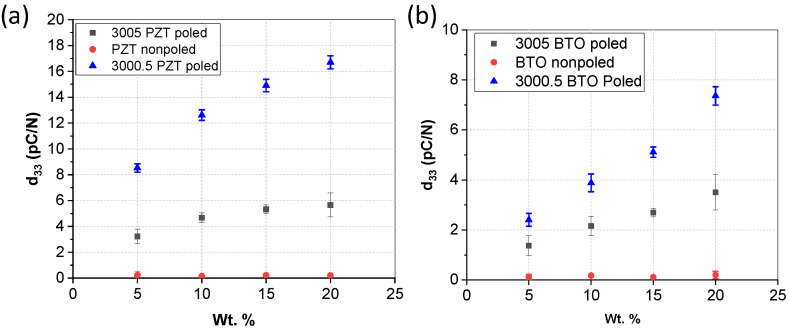
Piezoelectric measurements of the various SU-8 composite films as a function of concentration, (**a**) is PZT/SU-8 composites and (**b**) is for BTO/SU-8 composites.

The piezoelectric properties of the films (d_33,f_) are illustrated in Figure 10. The results illustrate that there were no piezoelectric properties prior to poling the films, but all composites had piezoelectric properties after poling. The piezoelectric properties increased as the concentration of the piezoelectric particles increased. The 3000.5 SU-8 composite films had a higher piezoelectric response, which is likely due to the alignment of the 0–3 composite. Thinner films will not suffer from alignment issues as the thickness of the film is on the same order as the diameter of the particles, so a sub-micron film may only have 1–2 layers of piezoelectric particles since the diameter of particles were a few hundred nm. However, thicker films produced from 3005 composite require alignment of the piezoelectric properties to maximize piezoelectric properties. The piezoelectric properties can be lower than in previous reports due to the testing, as we report on the d_33,f_ values that represent bulk film properties, whereas others have reported using piezoelectric force microscopy which measures individual grains [33,44]. The bulk property values are affected by the alignment and orientation of the films as individual grains could have opposite polarities that have a cancelling effect resulting in decreased bulk properties. In addition, PZT composite films demonstrated higher piezoelectric properties compared to BTO. The piezoelectric properties of a single layer of SU-8/PZT at 20 wt.% were comparable to those of traditional polymer piezoelectric thin films of PVDF [52].

### 3.3. Multiple Layer Composite Film Characterization

**Figure 11:**
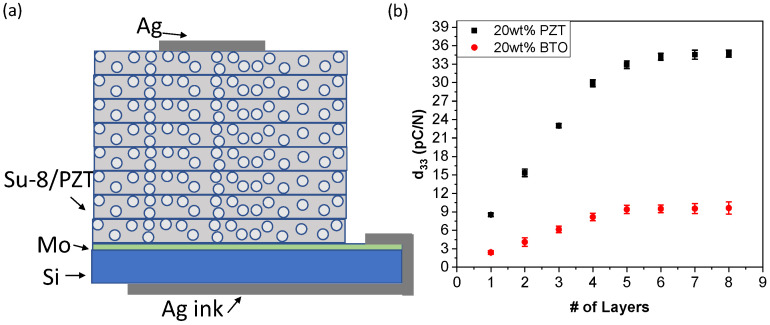
Multilayer piezoelectric composite stack structure made from 3000.5 SU-8, (**a**) schematic of an 8-layer SU-8/PZT stacked structure and (**b**) piezoelectric properties of SU-8 composite with multiple layers.

The ability to create thicker piezoelectric films is often needed to increase the piezoelectric properties and actuation or sensing capabilities. Although it was demonstrated that single layers of >12 μm could be produced, the piezoelectric properties decreased as a function of thickness due to the alignment issues of 0–3 composites. To overcome this, a stacked layer consisting of 3000.5 SU-8 composites was developed as shown in Figure 11a. The piezoelectric property results for the stacked layers are shown in Figure 11b. The results illustrate that as we increase the number of layers the effective d_33,f_ values increase by up to 4–5 layers. After 4–5 layers, the piezoelectric values saturate, which is likely due to alignment issues between stacked layers. The piezoelectric properties for PZT were significantly higher than those for the BTO composite. This result illustrates the potential to increase the piezoelectric properties by creating a stacked layer, but the alignment of the particles within the layers can significantly affect the results.

### 3.4. Integration of Composite Film into Device

**Figure 12:**
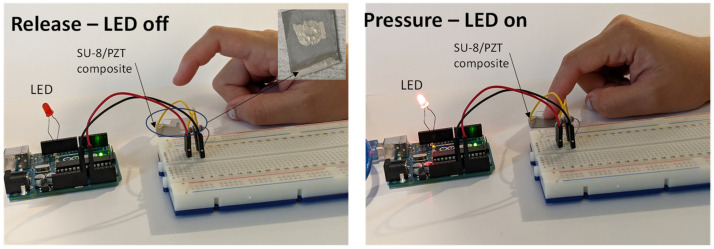
Demonstration of piezoelectric properties via tapping to light up an LED.

To demonstrate the piezoelectric properties, the SU-8/PZT film with 20 wt.% was connected to an Arduino sensor with an LED. The components of the device included a resistor to stabilize current, a piezo-component to generate a charge response, and an LED which was controlled via the Arduino code to turn on when there was a voltage readout of >5 V, which was caused by tapping the film. The results shown in Figure 12 demonstrates that the composite material had piezoelectric properties and was able to produce high voltage from simply tapping the film.

## 4. Conclusions

The microfabrication of 0–3 composites comprised of photopatternable SU-8 matrix and piezoelectric particles (PZT and BTO) was investigated for use in MEMS. The photolithography processing parameters were optimized to achieve a resolution < 2 μm using i-line exposure system. The reduced resolution is significantly lower compared to previous SU-8 piezoelectric composites which showed resolutions of 50–100 μm. Two different SU-8 matrix materials were investigated and thinned down to create SU-8 3005 and SU-8 3000.5 base material, which differed in density and % solids. The process parameters were optimized to increase manufacturability of the films and to make them compatible with standard microfabrication. The use of thinner helped reduce agglomeration. Using a speed mixer instead of a probe sonicator reduced the manufacturability time and increased homogenous mixture. The result was the ability to create low-resolution structures with a relatively high thickness of >12 μm for a single layer. Using fabrication techniques, we were also able to deposit structures on various substrates including glass and PDMS by adjusting the exposure dose.

The piezoelectric performance of the composites demonstrated a significant increase as a function of particle wt.%. The d_33,f_ values achieved for SU-8/PZT were comparable to PVDF, but the higher thermal properties of SU-8 make it more MEMS-compatible than PVDF due to its low Curie Temperature (approximately 100 °C). The study also investigated the potential of increasing thickness and piezoelectric properties by creating multiple stacked layers, which demonstrated significantly higher piezoelectric properties. However, alignment of the piezoelectric properties was a limiting factor that could be investigated in the future. The samples were prepared in a traditional lab environment, so there was no need for expensive cleanroom facilities, although yellow filter lights and an exposure tool were needed. This study demonstrated that SU-8 composite films that are MEMS compatible could be used to create new polymer piezoMEMS devices in the future.

## Data Availability

Data are contained within the article.
